# Shifting the paradigm in RNA virus detection: integrating nucleic acid testing and immunoassays through single-molecule digital ELISA

**DOI:** 10.3389/fimmu.2023.1331981

**Published:** 2024-01-03

**Authors:** Zhiyong Wang, Pei Wei

**Affiliations:** Department of Immunology, Zunyi Medical University, Zhuhai, China

**Keywords:** RNA viruses, nucleic acid testing, immunoassays, digital ELISA, single molecule detection

## Abstract

In this review article, we explore the characteristics of RNA viruses and their potential threats to humanity. We also provide a brief overview of the primary contemporary techniques used for the early detection of such viruses. After thoroughly analyzing the strengths and limitations of these methods, we highlight the importance of integrating nucleic acid testing with immunological assays in RNA virus detection. Although notable methodological differences between nucleic acid testing and immune assays pose challenges, the emerging single-molecule immunoassay-digital ELISA may be applied to technically integrate these techniques. We emphasize that the greatest value of digital ELISA is its extensive compatibility, which creates numerous opportunities for real-time, large-scale testing of RNA viruses. Furthermore, we describe the possible developmental trends of digital ELISA in various aspects, such as reaction carriers, identification elements, signal amplification, and data reading, thus revealing the remarkable potential of single-molecule digital ELISA in future RNA virus detection.

## Introduction

RNA viruses encompass a range of pathogens characterized by their use of RNA as a genetic material (1, 2). They include different types such as influenza virus (3, 4), Ebola virus (5, 6), and severe acute respiratory syndrome coronavirus 2 (SARS-CoV-2) (7, 8). Because of their high mutation rate and potential to cause severe diseases, they pose a significant threat to global public health. For this reason, they must be detected early to prevent disease transmission and facilitate effective treatments. Current detection methods include virus isolation and culture, viral sequencing, nucleic acid-based detection, and immunological-based detection (9, 10). Each method has unique advantages and challenges in terms of sensitivity, specificity, time efficiency, and resource requirements. Among them, nucleic acid- and immunological-based detection are the main methods for the early diagnosis of RNA viruses. Our viewpoint is that the integration of nucleic acid- and immunological-based approaches based on digital ELISA may be the future trend in RNA virus detection because it can minimize their respective limitations and maximize their advantages; thus, such integration can improve the accuracy and efficiency of RNA virus detection.

In this mini-review, we do not intend to provide an exhaustive survey of the field of digital detection because it has been extensively covered in the existing literature (11, 12). Instead, in the field of digital assay research, we focus on the detection of RNA viruses, specifically highlighting the convergence of nucleic acid testing techniques and immunoassays in the context of digital ELISA. We aim to offer an insightful yet succinct perspective on the revolutionary impact of digital ELISA technologies on the detection of RNA viruses.

## RNA viruses: understanding their characteristics and threats and the necessity for early rapid detection

RNA viruses, including double-stranded RNA viruses, single-stranded RNA viruses, and retroviruses, are a class of viruses that utilize RNA as their genetic material (1, 2). These viruses are characterized by their large size and short generation time; because their RNA-dependent RNA polymerase (RdRp) lacks a proofreading activity, they also have an extremely high mutation rate (13, 14). With a high mutation rate, RNA viruses in a single host can generate numerous genetic variations, which may result in viral phenotypes, including virulence and pathogenicity, thereby affecting the transmission and infection capacity of viruses (15, 16). They are capable of human-to-human transmission through various routes, including direct contact and inhalation; they can also be transmitted from humans to animals (17, 18). Because of the frequent recombination of genomes and the increased activity at the human–animal interface, RNA viruses are highly prone to causing new epidemics. In addition to their inherently high genetic diversity and rapid evolution, their transmission characteristics depend on their interaction with host immunity and the environment. The “survival path” of a newly emerging virus largely depends on the host’s immune response, and individuals with impaired adaptive immune responses more likely favor virus production and mutations (19, 20). During virus transmission, virus mutations may help evade previously existing immune responses and even change mechanisms that cross over with host cell pathways and are beneficial to infection; consequently, the probability of the emergence of new viral strains increases. This adaptive evolution, combined with the complexity of host immune responses, determines whether RNA viruses can cause localized, epidemic, or pandemic outbreaks (21, 22). In the past century, RNA viruses caused several major pandemics, posing significant threats to global health, and the COVID-19 pandemic in 2019 served as a stark reminder of this fact. Given the high frequency and widespread dissemination of RNA viruses, the detection and identification of these viruses are crucial for preventing potential pandemics and mitigating their societal impact. The clinical diagnosis of diseases caused by RNA virus infection primarily involves an integration of clinical symptoms, history of exposure to viruses, and virus detection. Because clinical manifestations and signs in infected patients are not entirely consistent, especially considering potential asymptomatic or mildly symptomatic infections, early viral detection is imperative for handling and controlling the large-scale transmission of RNA viruses.

## Advancements and limitations of RNA virus detection

Currently, detection or quantification methods for RNA viruses mainly include virus isolation and culture, nucleic acid-based detection methods, and immunological-based detection methods (9, 10). Among them, virus isolation and culture are the most reliable because they contribute to further analysis and characterization of the target virus (23, 24). However, most RNA viruses can be isolated only in laboratories with biosafety level 3 facilities. Additionally, virus isolation and culture are relatively time consuming, often requiring several days or even weeks (23, 24); therefore, they are unable to meet the demands for rapid diagnosis in most cases.

Nucleic acid-based RNA virus detection methods include sequencing, such as next-generation sequencing (NGS) (25, 26), and nucleic acid amplification-based detection technologies, such as isothermal and non-isothermal nucleic acid amplification (27–30). Sequencing technologies utilize extensive reference databases to explore new mutations and evolution of isolated RNA virus strains; determine the mutation rate of viruses and other related recombinants; and evaluate potential contact tracing, virus evolution research, and molecular epidemiology (25, 26). However, sequencing analyses require highly skilled personnel to operate expensive instruments under strict laboratory conditions. They are time-consuming and relatively costly, severely limiting their widespread application in RNA virus detection (31). By comparison, detection technologies based on nucleic acid amplification feature high sensitivity, specificity, speed, quantifiable results, and automation; therefore, they are suitable for large-scale screening in the early stages of an epidemic and have become the most widely accepted RNA virus diagnostic method (25–28). However, they are also limited in terms of experimental conditions, equipment costs, personnel costs, and detection cycles. For instance, sample contamination caused by factors such as aerosols during nucleic acid amplification negatively affects detection accuracy (32). These limitations, combined with the inherent instability of RNA molecules, further restricts the application of such detection technologies in large-scale screening for early-stage RNA virus infections.

Immunological detection strategies utilize antibody–antigen reactions to detect target viral proteins (antigens) in samples or antibodies against viruses in serum (33, 34). An example of such strategies is enzyme-linked immunosorbent assay (ELISA) (35, 36). Immunoassay-based detection methods are cost effective, rapid, and simple, making them suitable for wide screening. However, their widespread application in early stages is restricted because of some inherent limitations. First, immunological detection has lower sensitivity than nucleic acid amplification techniques and may produce false-negative results in cases of low viral loads or limited serum antibody concentrations. Second, for viral antigen detection, the preparation and production of corresponding detecting antibodies require time, impeding the urgent demand for large-scale early screening. Third, the occurrence of cross-reactivity in antigen–antibody reactions may lead to false-positive results, thus limiting the accuracy of results to some extent (37).

Nucleic acid and immune detection techniques are the two main methods for the early screening of RNA virus infections, each with their own unique advantages and inherent limitations. As such, these methods should be integrated into a unified approach to form a more comprehensive and effective diagnostic system. Because of the fundamental methodological differences between nucleic acid testing and immune testing, they have a considerable gap. By comparison, ELISA exhibits strong compatibility, in addition to its numerous advantages, such as simplicity, practicality, speed, and reasonable cost; thus, it offers the potential for achieving technical integration between nucleic acid testing and immune testing.

## Digital ELISA: a bridge integrating nucleic acid testing and immune detection methods

Immunological assays have evolved over years of development, leveraging their exceptional compatibility to be integrated with various interdisciplinary fields; as a result, a series of noteworthy technological innovations has been developed. These advancements are evident in several aspects of immunological assays, including reaction carriers, recognition elements, signal amplification, and reading methods. Of particular importance is the fusion of single-molecule detection technology and immunological assays, resulting in single-molecule counting immunity, which is also known as digital ELISA (38, 39). This revolutionary technique has overcome the sensitivity limitations of traditional immunological assays, thus offering an extraordinary level of sensitivity and high resolution that macroscopic measurements have failed to achieve.

**Figure 1:**
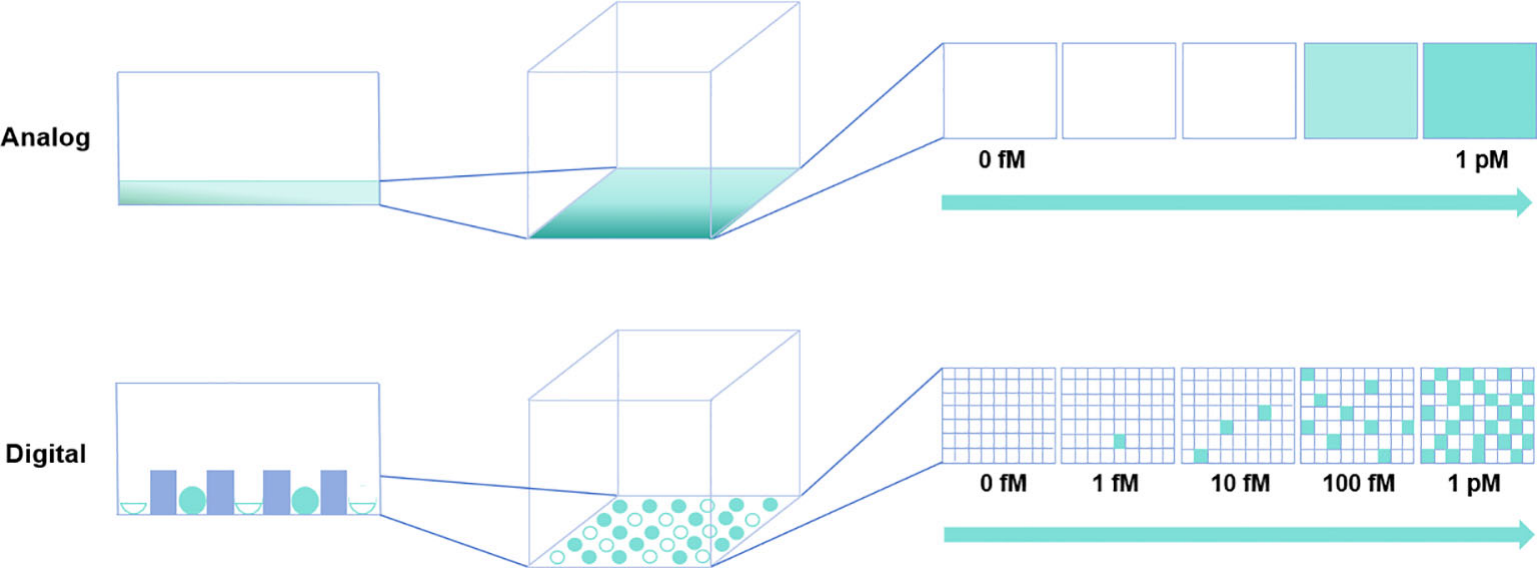
Comparative schematic of traditional versus digital bioassays. In the traditional (analog) assay depicted in the upper panel, rapid diffusion of reaction products is suboptimal for detecting highly diluted target molecules. Conversely, in the digital bioassay shown in the lower panel, reaction products are compartmentalized within microchambers, enabling detection at the single-molecule level.

The pursuit of improving the detection limit of biomolecules has given rise to the trend of digital measurement. The term “digital” in computing refers to the binary code of 0 s and 1 s. Similarly, in detection systems, the switching of specific signals of biomolecules between discrete states (such as presence or absence, occurrence or non-occurrence, and binding or non-binding) is also a binary event. Unlike traditional bioassays typically conducted in a single reactor, in digital assays, individual target molecules are randomized into microchambers (11, 12). Once the signal separation is established between positive microchambers with a single target molecule and negative microchambers without the target molecule (reaction microchambers display binary discrete signals representing “1” or “0”), the absolute quantity of target molecules can be determined by counting positive reactors after digitizing signals with sufficient thresholds (11, 12). **Figure 1** provides a schematic diagram comparing digital bioassays and traditional bioassays. In digital bioassays, the reaction in each microchamber displays a positive or negative signal, meeting the prerequisite conditions for a binomial trial. Therefore, digital bioassays should use the Poisson distribution, which is a limiting case of the binomial distribution (11, 12). Unlike the continuous signal recording noted in traditional ELISA, analysis is performed by recording discrete signals in digital ELISA. This strategic deviation circumvents the inaccuracies introduced by macro-level signal fluctuations, thereby providing an overwhelming advantage for detecting target molecules at low concentrations. In fact, for the same target molecule, the detection limit of digital assays is generally at least 1/1000 that of conventional assays (39, 40).

Digital biodetection systems consist of three main components: microchamber reactors for microsegmentation, markers for studying or labeling target molecules, and substrates or chemical probes for providing detectable signals (11, 12). Depending on the type of signal amplification in the microreactor, digital assays for RNA viruses can be divided into two categories. The first category includes digital PCR and other digital nucleic acid amplification assays, which rely on the exponential amplification of viral RNA transcribed into cDNA. However, the non-linear enzyme-catalyzed reverse transcription process introduces quantitative bias (41, 42). Moreover, the contamination of samples during the nucleic acid amplification process greatly affects the accuracy of quantitative results (32). Therefore, digital detection methods based on non-reverse transcription and non-nucleic acid amplification can be more effective for the quantification of RNA viruses. Unlike the first category involving digital PCR, which relies on the amplification of the target molecule, the other category of digital detection of RNA viruses relies on the amplification of detection signals (such as enzymes and fluorescent probes); one such example is digital ELISA.

**Figure 2:**
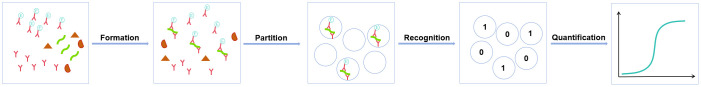
Schematic outline of the digital ELISA process for detecting viral RNA. Viral RNA is captured by specific antibodies (or nucleic acid aptamers or molecular polymers) to form an immune complex, which is then compartmentalized into discrete, countable units. Quantification of individual immunocomplex molecules is achieved by recognizing the signal generated by the marker molecules conjugated to the antibodies.

The principal methodology of digital ELISA involves a four-step process: the formation of immune complexes to capture target molecules (formation), spatial or temporal partition of these complexes to make them distinguishable and quantifiable (partition), recognition of signals generated by the target molecules through optical or electrical methods (recognition), and absolute quantification using the Poisson distribution (quantification) (12, 43). Advances in the development of RNA antibody mimetics (44, 45), nucleic acid aptamers (46, 47), and molecularly imprinted polymers (48, 49) have made it feasible to directly detect trace amounts of viral RNA using digital ELISA; this is a significant breakthrough. This capability implies that high-sensitivity RNA virus detection can be performed even without the need for a nucleic acid amplification step, thereby reducing the assay time and completely bypassing issues related to sample contamination that can occur during amplification. This ultimately reduces the stringent environmental controls required for nucleic acid assays and has profound implications for the real-time detection of RNA viruses. **Figure 2** provides a schematic representation of the use of digital ELISA for the direct detection of viral RNA. Currently, successful commercial digital ELISA detection platforms, such as Simoa (50) and Erenna (its upgraded version is called SMCxPro) (51), are widely used for practical applications. With these commercial digital ELISA technology platforms, quantitative analysis of antibodies (IgG, IgM, and IgA) in the blood of SARS-CoV-2 infected individuals can be performed on the same day when the symptoms first appear and the nucleic acid test yields positive results, with a sample volume of less than 1 μL (52). Most excitingly, research utilizing the Simoa platform has demonstrated the capability to detect SARS-CoV-2 RNA in saliva, achieving a remarkable detection limit of 3.4 fM (53).

Currently, digital ELISA is undergoing rapid development; for example, the widespread application of droplet discretization method has achieved the full loading of immunocomplexes (54, 55). Scholars such as Akama proposed a soft discretization method using enzyme-catalyzed deposition, which is expected to avoid the dependence on complex droplet generation equipment (56). Other researchers developed a particle diffusion recognition method, which allows particles to exhibit characteristic Brownian motion in a limited space; in this way, uniform immunoanalysis can be digitized without washing (57, 58). Chen and others visualized single immunocomplexes by using microbubbles produced through hydrogen peroxide catalysis with platinum nanoparticles (59). Zhang and others used the enhancement effect of microsphere lenses to visualize single target labels successfully under a 20× objective (60). Furthermore, emerging evidence suggests that digital ELISA will make further breakthroughs in portability, readiness, simplicity, high throughput, and ultrasensitivity (61, 62).

## Navigating the future of RNA virus detection: the potential of digital ELISA

Because of factors such as testing costs, efficiency, reagent stability, and maintenance costs, the large-scale application of digital ELISA for early RNA virus detection remains impractical. However, looking back at the entire history of diagnostic technology development, the improvement of any detection technique requires a long period of exploration. The significance of digital ELISA in RNA virus detection lies in its ability to integrate nucleic acid and immune detection, rather than just improving existing detection techniques. Equally important is the powerful compatibility of ELISA, which provides a wide range of options for further expanding RNA virus detection.

For example, in the optimization of reaction carriers, digital ELISA can be combined with paper-based biosensors (63, 64) to develop shorter, lower-cost, and field-deployable paper-based detection systems that meet the needs of large-scale, real-time RNA virus detection. With the development of low-cost data reading devices, such as desktop scanners or mobile cameras (65), RNA viruses can be detected early in resource-limited areas on a large scale. In terms of recognition element optimization, given that antibodies as recognition molecules have limitations, such as poor stability, high costs, and difficulty in large-scale production, digital ELISA can be combined with nucleic acid aptamers (46, 47) or molecularly imprinted polymers (48, 49) to eliminate dependence on antibodies. For RNA viruses, these alternatives further remove barriers to the integration of nucleic acid and immunoassays. In the aspect of signal amplification optimization, various nanomaterials, such as nanospheres (60, 66), nanopores (67), upconversion nanoparticles (68, 69), and quantum dots (70, 71), when combined with digital ELISA, can greatly enhance the amplification of collected signals. Additionally, the combination of CRISPR-Cas system with traditional ELISA can greatly enhance the detection sensitivity of the latter (72). Therefore, the effect produced by the combination of CRISPR-Cas system with digital ELISA is worth anticipating. The development of these methods ensures the sensitivity of digital ELISA, compensating for the loss of sensitivity because of avoiding nucleic acid amplification in the unification of nucleic acid and immunoassays. In terms of reading methods, neither fluorescence scanning after enzyme-catalyzed signal amplification in the Simoa detection system (50) nor device-dependent single-molecule signal scanning in the Erenna (SMCxPro) system (51) is suitable for early large-scale screening of RNA viruses. However, through explorations such as bright-field or dark-field imaging, digital ELISA is expected to achieve smartphone-based reading methods (65). Particularly, it can be applied to potentially integrate digital reading content with cutting-edge technologies, such as artificial intelligence (AI), machine learning (ML), the Internet, and the Internet of Things (IoT). By integrating AI and ML into diagnostics, we can achieve unprecedented analysis speed and accuracy. For example, these technologies can screen large amounts of patient data, identify patterns and trends that may indicate an outbreak, and even predict epidemics before they reach pandemic levels. In addition, the integration of digital ELISA with the Internet and IoT can revolutionize remote healthcare. Virus detection can be performed in real time, and data can be transmitted immediately to healthcare providers regardless of their geographical location. Thus, response times can be remarkably accelerated, and more targeted interventions can be implemented, especially in rural or underserved areas. However, these processes must be built on robust privacy and cybersecurity measures to protect sensitive health data.

## Conclusion

Although the development of digital ELISA faces numerous challenges, its potential advantages are remarkable. This technology may be used to technically integrate nucleic acid testing and immunoassay, thereby transforming the paradigm of RNA virus detection and improving speed, accuracy, and diagnostic range relative to those of current methods. The exceptional sensitivity and specificity of digital ELISA can enhance the early detection of viruses; consequently, therapeutic responses will be faster and more targeted, considerably mitigating the spread and severity of diseases. Addressing the high mutation rates of RNA viruses, digital ELISA has a rapid detection capability and can serve as an efficient and effective tool, thereby identifying and possibly preventing the spread of emergent and recurrent infections. Therefore, the advancement of digital ELISA technology promises to revolutionize RNA virus detection.

## Author contributions

ZW: Writing – original draft. PW: Writing – review & editing.
